# Comparing the efficacy of Morphine alone with Morphine and mgso4 in pain management after coronary artery bypass surgery

**DOI:** 10.12669/pjms.342.14280

**Published:** 2018

**Authors:** Rana Altaf Ahmad, Syed Suhail Ahmad, Waqas Hamid, Aamir Furqan

**Affiliations:** 1 Dr. Rana Altaf Ahmad, MBBS FCPS. Department of Anesthesia, Chaudhry Pervaiz Elahi Institute of Cardiology (CPEIC), Multan, Pakistan; 2 Dr. Syed Suhail Ahmad, MBBS, FCPS. Department of Anesthesia, Chaudhry Pervaiz Elahi Institute of Cardiology (CPEIC), Multan, Pakistan; 3 Dr. Waqas Hamid Khan, MBBS, FCPS. Department of Cardiac Surgery, Chaudhry Pervaiz Elahi Institute of Cardiology (CPEIC), Multan, Pakistan; 4 Dr. Aamir Furqan, MBBS, FCPS. Department of Anaesthesia, Nishtar Medical University, Multan, Pakistan

**Keywords:** Morphine, Mgso4, CABG, Pain

## Abstract

**Objective:**

To compare the effectiveness of Morphine alone and Morphine with MgSo4 in pain management after CABG surgery.

**Methods:**

This randomized control trial was conducted in the department of anesthesia and critical care Choudhary Pervaiz Ellahi Institute of Cardiology, Multan from November 2016 to June 2017. All collected data was entered and analyzed by using computer software SPSS version 23.1. Quantitative data like age, VAS score was analyzed and presented as mean and standard deviation. Similarly qualitative data like gender and ASA status was calculated and presented as frequency and percentages. Independent sample T-test was applied for significance of VAS score. P value ≤0.05 was considered as significant.

**Results:**

A total number of 150 patients of both genders were included in this study. The main outcome variables of our study were VAS score. It was observed that, in group (M), the mean VAS score after 4, 12 and 24 hours of operation was 5.24±1.61, 5.8±2.27 and 5.44±2.27 respectively. And in group (MM), the mean VAS score after 4, 12 and 24 hours of operation was 4.36±2.58, 3.48±2.10 and 4.12±1.05 respectively. It was noted that both groups had statically significant difference of VAS score, as group (M) had higher VAS score than group (MM).

**Conclusion:**

Morphine with Mgso4 has better efficacy as compared to morphine alone when used as analgesic agent after CABG surgery.

## INTRODUCTION

Pain can be defined as unusual sensory and emotional feeling due to tissue damage.^1^ Pain is a part of human life and varies from low intensity to high intensity. Many techniques and attempts have been made to relieve pain since the time mankind was created. Among different types of pain, pain after surgery is a major unhappy event for patients.^2^ Patients are always in a state of fear due to pain and unhealthy feeling during any type of major surgery. Pain complaints of patients should be relieved as soon as possible.^3^ However, sometimes patients complain about uncontrolled pain may be due to insufficient analgesia provided by the doctor or negligence of the staff.^4^,^5^ Things that prevent a doctor to administer proper analgesia are patient addiction, drug intolerance and adverse effects. These all factors are also a hurdle for prescribing opioid analgesics.^6^ Inadequate pain relief in youngsters is a challenge (age between 2 to 14 years).

In 1984 James Moore used opium for the first time in post operative cases as a pain relieving agent.^7^ He clearly justified the use of opium in post operative period as it provides good analgesia with adequate sleep and control over irritation.^8^ A large number of pain killers (opioids or non opioids) are available now but for the management of acute pain World Federation of Societies of anesthetist (WASA) has been made, and it’s role clearly defined for worldwide issues on pain management.^9^ WASA designed a ladder to control pain after surgery with injectable opioids combined with local blocks and other drugs. According to the protocol, pain slowed down with passage of time and duration operation time.^10^ Medications were weaned off from high doses to low doses, than oral medication (non steroidal anti inflammatory drugs). It is observed that intensity of pain and effect of opioids vary person to person, so before selecting the drugs patient should be assessed for intensity of pain and drug addiction history.^11^,^12^

After lot of modalities and drugs inventions a large number of patients still suffered with severe pain in post operative period. Aim of our study was to compare the two drugs regimes Mgso4 and Morphine with Morphine alone to control in post cardiac surgery period, there is no local study available on this topic so our study will be a local reference in near future.

## METHODS

After the approval of the ethical committee of the hospital, the total number of 150 patients was included in the study through non probability consecutive sampling. Sample size was calculated by using online source Openepi.com. Patients of both sex who belonged to ASA physical status III, IV and selected for CABG surgery were included. The protocol, procedure and purpose of study were completely explained to patient as well as written consent was taken. All the patients were randomly allocated to the Morphine and Morphine with Mgso4 group by lottery method. Patients with impaired hepatic and renal function, myopathy, diabetes, hypertension, neurological disorder, pregnant women, obese patients (body mass index more than 28kg/m) and patients who were taking magnesium and calcium channel blockers, any kind of cardiac surgery except CABG, known history of allergy to the drugs used during surgery, patients with history of drug abuse or chronic analgesic use and ejection fraction less than 40% were excluded. All the routine check up and the complete pre- anesthesia investigation of all the patients were done. As premedication, one hour before planned surgery, ranitidine 150mg and midazolam 0.05mg/kg were given to all patients. When patients were shifted to the operation theatre, the baseline value of diastolic blood pressure (DBP), heart rate (HR), systolic blood pressure (SBP) and oxygen saturation were recorded. After proper intravenous approach in all patients, anaesthetic solution was given to patients.

Effectiveness was measured in terms of mean VAS score in both groups. Patients were ventilated with 100% oxygen initially and then put on ventilator; intermittent positive airway pressure mode was used for ventilation during surgery and maintenance of anesthesia. For the maintenance of anesthesia 50% air, 50% oxygen, Morphine 0.1-0.2 mg/kg/h, propofol 50 μg/kg/min and muscle relaxant atracurium 0.5 mg/kg/h was used in group M. but 4g Magnesium sulphate intravenous given additionally as above protocol when separated from CPB in group MM. After surgery patients were shifted to ICU department of hospital on same ventilator support. About four hour after shifting when weaning started and removal of endotracheal tube VAS score was measured.

However, this randomization was done by closed envelope method. Two groups were labeled as Group M morphine and Group MM magnesium sulphate and morphine. Magnesium sulphate used was basically preservative free. Preoperatively patients were explained the visual analogue pain scale (VAS) scores. It was started to be recorded after four hours of surgery and post-operatively upto 24 hours. When VAS score was > 3 the analgesic medicines were given to the patients. Analgesia duration was recorded from 1^st^ analgesic injection to the first pain complain, or a reported VAS>3.

Collected data was entered and analyzed by using statistical software SPSS version 23, mean and standard deviations were calculated for quantitative variables like age and VAS score. Similarly frequency and percentages were calculated for qualitative variables like gender. Independent sample t-test was applied to check the significance of results and p≤ 0.05 was considered as significant.

**Fig.1:**
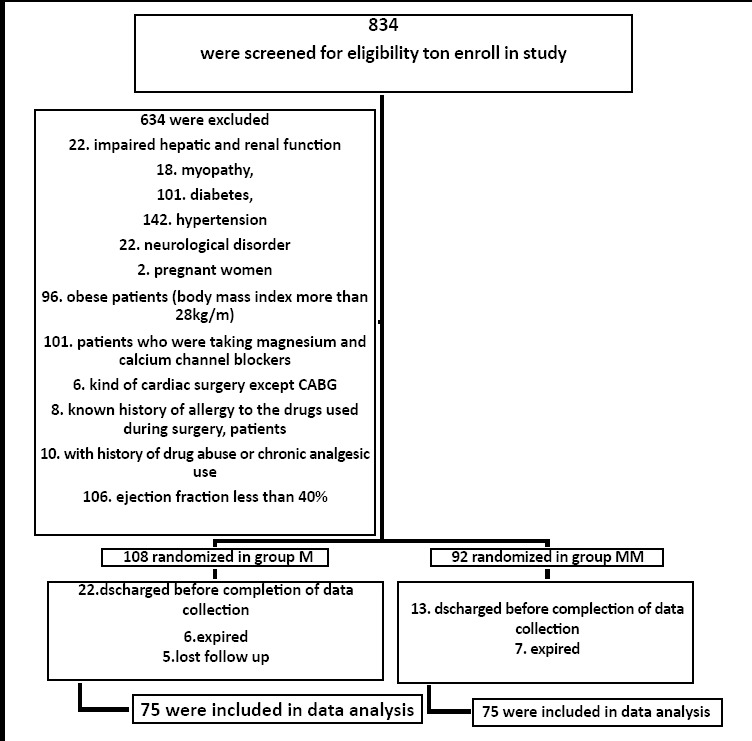
Flow of Study Patients.

## RESULTS

A total number of one hundred fifty patients were included in this study. Gender distribution showed that there were more males than females i.e. 65.3% (n=98) and 34.7% (n=52) respectively. The mean age of the patients was 60.28±6.54 years. It was observed that out of 100% (n=150) patients, 57.3% (n=86) belong to ASA 3 and 42.7% (n=64) ASA 4(Table-I).

**Table-I:** Demographic Variables: (n=150).

Characteristics	Frequency	Percentage (%)	Test of Sig.
**Gender**			
Male	98	65.3	t=-0.830
Female	52	34.7	p=0.408
Total	150	100.0	
**Stratified Age**			
37-55years	40	26.7	t=2.276
56-73 years	110	73.3	p=0.024
Total	150	100.0	
**Stratified BMI**			
22-28	216	56	t=-0.698
29-36	170	44	p=0.486
Total	150	100.0	
**ASA**			
ASA 3	86	57.3	t=-1.092
ASA 4	64	42.7	p=0.277
Total	150	100.0	
**Descriptive Statistics**		
**Mean±S.D**			
Age	60.28±6.54 years		

These patients were divided into two groups, 75 in each, i.e. Group (M) and group (MM). The mean age of the patients in group (M) was 59.63±8.34 while the mean age of the patients in group (MM) was 60.94±3.97. Gender distribution showed that, in group (M), there were 64% (n=48) and 36% (n=27) males and females respectively while in group (MM), there were 66.7% (n=50) and 33.3% (n=25) males and females respectively.

The main outcome variables of our study were VAS score. It was observed that, in group (M), the mean VAS score after 4, 12 and 24 hours of operation was 5.24±1.61, 5.8±2.27 and 5.44±2.27 respectively. And in group (MM), the mean VAS score after 4, 12 and 24 hours of operation was 4.36±2.58, 3.48±2.10 and 4.12±1.05 respectively. It was noted that group (M) had higher VAS score than group (MM). When patients were stratified into different age groups, it was noted that majority of patients i.e. 73.3% (n=110) were aged from 56 to 76 years while only 26.7% (n=40) patients were aged from 37 to 55 years (Table-I).

No significant difference was found between VAS score with gender (P=0.408), ASA (p=0.277), and stratified BMI (P=0.486) except stratified age (P=0.024). To check the equality of the means of VAS score in two groups i.e. group (M) and group (MM), independent samples t-test was applied. It was seen that the mean VAS score for group (M) i.e. 5.49±2.06 was statistically significantly different from the mean VAS score for group (MM) i.e. 3.98±2.02 with p-value 0.000 (Table-II).

**Table-II:** VAS score in both groups after treatment.

Groups	Time	Mean ± S.D (VAS Score)	Test of Sig.
Group (M)	After 4 hours of operation	5.24±1.61	t=4.516
	After 12 hours of operation	5.80±2.27	p=0.000
	After 24 hours of operation	5.44±2.27	
Group (MM)	After 4 hours of operation	4.36±2.58
	After 12 hours of operation	3.48±2.10	
	After 24 hours of operation	4.12±1.05	

## DISCUSSION

Cardiac surgery is a stressful event in life of a person and pain control after cardiac surgery is a challenge for health care professionals. Sometimes, pain after surgery is a bad experience throughout the life of a person.^13^ Many studies have been done specifically on pain control after surgery. In our study we evaluated the role of drugs after heart surgery in this study, we assessed pain at four different stages: at one hour of endotracheal Extubation, at 4 hours of operation, after 12 of operations and after 24 hours of operations. Highest intensity of pain was noticed at one hour of extubation and minimum pain was observed after 4 hours of operations and maximum doses were also given in this period.

PE Ozcan a Turkish anesthetist conducted a study on role of magnesium sulphate in postoperative pain management.^14^ He compared mgso4 with morphin in post operative pain relief in thoracotomy patients and concluded that there was no significant difference in demographics and mean VAS score in both groups. Results of study were as. These findings are also comparable with our findings.

In December 2008 Ferasatkish R et al.^15^ conducted a study on efficacy of Mgso4 in pain control after CABG surgery. He compared Mgso4 with placebo, outcomes of his study were extubation time and pain relief. He reported that Mgso4 group have less extubation time and pain score was also less in Mgso4 group than placebo when recorded at 12^th^, 18^th^, and 24 hours after cardiac surgery.

Arikan M et al.^16^ conducted a study on Mgso4 and ketamine on postoperative pain control and morphine consumption. In his study he reported that mgso4 is less effective than Ketamine and morphin consumption is also high in post operative patients. Results of this study were identical to our findings but in our morphine consumption was not followed.

Nadri S et al.^17^ conducted a study on Mgso4 efficacy of pain control after varicicelectomy. He used 2 mg mgso4 diluted in 2 ml normal saline and injected after surgery immediately and 2^nd^ group received 2ml normal saline only. Results of his study revealed that mgso4 group have less vas score 0.91 as compared to control group 1.30. This study can also be compared with our results.

Magnesium has been used in cardiology and anesthesia for many years as antiarrythmic and anticonvalsant drug but its role as pain relief is not clear as yet.^18^ Some studies have declared its role as analgesic by acting as a NMDA antagonist which controls pain by inhibiting sensitization of nociceptive. Some studies justify its role as an agent which reduced the release of chaticholamine with sympathetic stimulantion. Data from previous studies goes in the favour of NMDA receptor antagonist when administered in low doses. At low doses it suppress the pain threshold.^19^, ^20^

In a study conducted by Lakdizaji S^21^ it was reported that after cardiac surgery background infusion of morphine improves the pain relief with minimum adverse effect that it increase the morphine consumption. In another study by Guler T et al^22^ also reported that ideal pain management can be achieved with continuous morphine infusion after percutaneous coronary angiography. It will improve the pain control and leads to the better outcomes after surgery. Similar findings were also reported by Mota FA et al^23^ and Dal D et al^24^ that morphine is a drug of choice or pain management especially in heart surgeries (after PCA or CABG). In our study we also concluded that morphine is a better drug for control of pain than but it is more efficacious when Mgso4 was added in ICU patients.

## CONCLUSION

Our clinical trial showed that morphine with Mgso4 has better efficacy as compared to morphine alone when used as analgesic agent when VAS score was measured after every four hours till 24 hours after CABG surgery.

### Authors’ Contribution

**RA** conceived, designed, did statistical analysis & editing of manuscript.

**SA and WH** did data collection and manuscript writing.

**AF** did review and final approval of manuscript.
